# SG-APSIC1098: Impact of the COVID-19 pandemic on influenza vaccination uptake among healthcare workers

**DOI:** 10.1017/ash.2023.20

**Published:** 2023-03-16

**Authors:** Min Yi Gwee, Lim John Wah, Indumathi Venkatachalam, Jean Sim Xiang Ying, May Kyawt Aung, Yang Yong, Conceicao Edwin Philip, Shalvi Arora, Aung Myat Oo, Shawn See Wee Jin

**Affiliations:** Singapore General Hospital, Singapore; Singapore General Hospital, Singapore; Singapore General Hospital, Singapore; Singapore General Hospital, Singapore; Singapore General Hospital, Singapore; Singapore General Hospital, Singapore; Singapore General Hospital, Singapore; Singapore General Hospital, Singapore; Singapore General Hospital, Singapore; Singapore General Hospital, Singapore

## Abstract

**Objectives:** Influenza vaccination is encouraged for all healthcare workers (HCWs) to reduce the risk of acquiring the infection and onward transmission to colleagues and patients during the influenza season. Thus, vaccination was introduced at Singapore General Hospital (SGH) in 2007 and has been offered to all HCWs at no cost. The HCW influenza vaccination program is conducted annually in October and biannually during years with vaccine mismatch. However, influenza vaccine uptake remained low among HCWs. We sought to determine the impact of the coronavirus disease 2019 (COVID-19) pandemic on influenza vaccine uptake among HCWs. **Methods:** At SGH, 2 methods of vaccine delivery are offered: centralized (1-month drop-in system during office hours) and decentralized (administered by vaccination teams in offices or ward staff in inpatient locations). In the 4-year study period between 2018 and 2021, 6 influenza vaccination exercise campaigns were conducted during 8 influenza seasons. During each exercise, ~9,000 HCWs were eligible for vaccination. **Results:** Prior to the COVID-19 pandemic, vaccine uptake in the Southern Hemisphere was 77.6% (6,964 of 8,977) in 2018 and 84.2% (7,296 of 8,670) in 2019. During the COVID-19 pandemic in 2020, vaccine uptake in the Southern Hemisphere increased by 10% to 94.1% (8,361 of 8,889). In the Northern Hemisphere, vaccine uptake was 79.2% (7,114 of 8,977) in 2018, and this increased by 17.9% to 97.1% (8,926 of 9,194) during the COVID-19 pandemic in 2020. During the 2021 Southern Hemisphere influenza season, no vaccination program was conducted because the risk of influenza was considered low due to the closure of international borders and the implementation of public health measures. In addition, priority was given to COVID-19 vaccination efforts. **Conclusions:** Increased uptake of the influenza vaccination was observed during the COVID-19 pandemic. Anxiety created by the respiratory disease pandemic and debate surrounding vaccines likely contributed to increased awareness and uptake in influenza vaccine among HCWs.

